# Neuro-Immune Circuits Regulate Immune Responses in Tissues and Organ Homeostasis

**DOI:** 10.3389/fimmu.2020.00308

**Published:** 2020-03-20

**Authors:** Manuel O. Jakob, Shaira Murugan, Christoph S. N. Klose

**Affiliations:** ^1^Department of Microbiology, Infectious Diseases and Immunology, Charité - Universitätsmedizin Berlin, Berlin, Germany; ^2^Group of Visceral Surgery and Medicine, Department of BioMedical Research, University of Bern, Bern, Switzerland

**Keywords:** neuro-immune interactions, chronic inflammatory diseases, autonomous nervous system, enteric nervous system (ENS), tissue homeostasis

## Abstract

The dense innervation of the gastro-intestinal tract with neuronal networks, which are in close proximity to immune cells, implies a pivotal role of neurons in modulating immune functions. Neurons have the ability to directly sense danger signals, adapt immune effector functions and integrate these signals to maintain tissue integrity and host defense strategies. The expression pattern of a large set of immune cells in the intestine characterized by receptors for neurotransmitters and neuropeptides suggest a tight neuronal hierarchical control of immune functions in order to systemically control immune reactions. Compelling evidence implies that targeting neuro-immune interactions is a promising strategy to dampen immune responses in autoimmune diseases such as inflammatory bowel diseases or rheumatoid arthritis. In fact, electric stimulation of vagal fibers has been shown to be an extremely effective treatment strategy against overwhelming immune reactions, even after exhausted conventional treatment strategies. Such findings argue that the nervous system is underestimated coordinator of immune reactions and underline the importance of neuro-immune crosstalk for body homeostasis. Herein, we review neuro-immune interactions with a special focus on disease pathogenesis throughout the gastro-intestinal tract.

## Introduction

Immune responses at mucosal barriers are of particular interest because the mucosa is the primary entry port for many pathogens as well as the major site for chronic and sometimes detrimental immune responses. The interaction between the immune and the nervous system at mucosal barriers is attracting more attention from researchers worldwide. Recent advances in understanding the role of the interplay between both systems have uncovered a pivotal role of the nervous system in modulating immune responses and vice versa. The notion that the immune system and the nervous system share many commonalities emerged the idea of strong cross-interactions (1). Evolutionary similarities, such as signaling via transmitters, information delivery to distant body regions and migratory behavior, link the nervous with the immune system and together they coordinate the integration of danger signals to external environmental stimuli (1). The nervous system *per se* is a large interface that is strongly involved in maintaining body homeostasis (2). On the one hand, autonomic neurons sense a broad variety of parameters such as mechanical distortion, physicochemical attributes, secretions, nutrients and toxins (3). On the other hand, the autonomic nervous system controls effector functions such as intestinal motility, blood flow, and secretory functions (4). Specifically, the enteric nervous system (ENS) controls and dictates the motor function of smooth muscle cells throughout the gastrointestinal tract. Such coordinated muscular activity results in squirting of ingested food, allows for mixing with digestive enzymes and eventually commands the aboral transport of non-digestible products (5). Of note, the intestine is densely innervated by the autonomous nervous system and populated by hematopoietic cells, therefore providing opportunities for neuro-immune interactions (4). Advances in the understanding of neuro-immune interactions has uncovered the immune-modulatory properties of neurons and emerged an interesting treatment approach for inflammatory conditions (6).

Current treatment modalities for autoimmune diseases, such as inflammatory bowel diseases (IBD), are often insufficient. These therapies target specific molecules on the surface of immune cells or, more general, dampen immune responses. However, this strategy fails to control disease-activity in many patients (7). There is strong evidence that modulation of the autonomic nervous system can exert strong anti-inflammatory effects, even after exhausted therapeutical modalities (8, 9). Current biologics targeting immune cells for example in IBD are often insufficiently effective and associated with severe side effects (10). There is a strong need for novel therapeutics with low side effects that have immune-modulatory functions rather than solely dampening effector functions. Thus, treatment strategies that harness neuro-immune interactions may be a promising approach because it is known that the nervous system is able to exert strong anti-inflammatory effects in mice and humans (9, 11). Herein, we review current knowledge in neuro-immune-interactions that maintain body homeostasis with a special focus on disease entities and the translational relevance as a potential therapeutic target in inflammatory diseases within the intestine.

## Anatomical Organization of the Autonomous Nervous System

**Figure 1:**
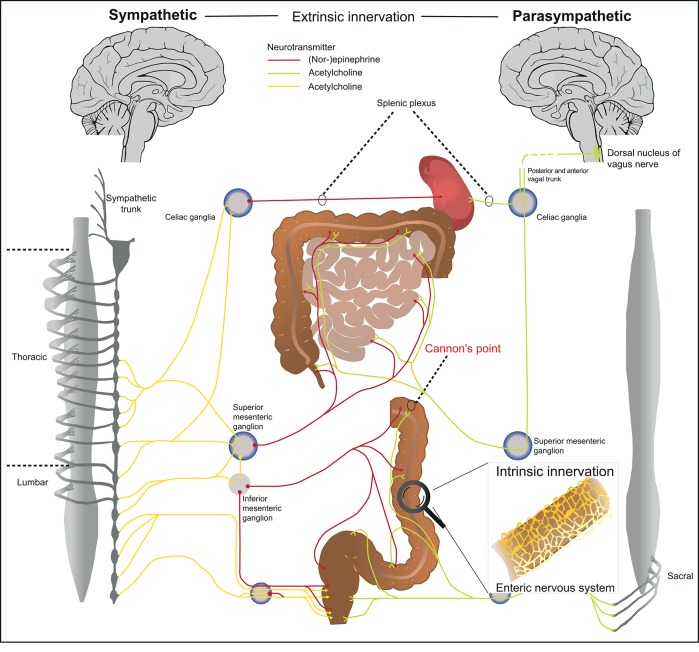
Anatomical organization of the autonomous nervous system. The autonomous nervous system is organized in three anatomical and biochemical distinct systems. (1) The sympathetic nervous system has its preganglionic cell bodies in the thoraco-lumbar region (sympathetic trunk). The pregangliotic sympathetic neurons synapses with the postgangionic neuron in the sympathetic trunk, whereas the long postganglionic neuron (red) innervates the respective part of the gastro-intestinal tract. (2) The cell bodies of the parasympathetic nervous system are located in the brainstem and the pelvic sacral nerves. The vagal nerve includes preganglionic fibers from parasympathetic nervous system (green) that innervates the gastro-intestinal tract and ends just before the splenic flexure of the transverse colon (also known as Cannon's point). After Cannon's point, the colon is innervated by the pelvic sacral plexus. The postganglionic neuron is localized in immediate proximity to the target organ. (3) The enteric nervous system is located within intestinal tissues (Auerbach plexus, Meissner plexus) and has a characteristic architecture (details see Figure 2).

**Figure 2:**
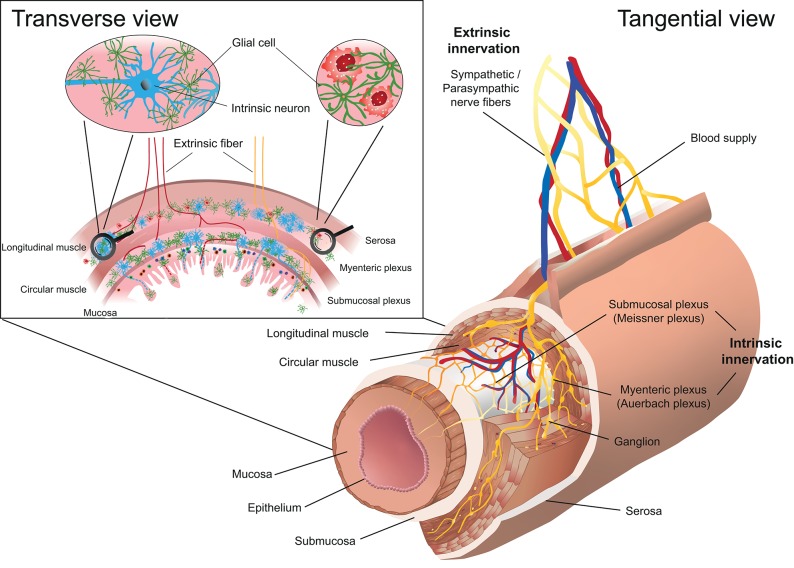
Schematic representation of the enteric nervous system (ENS) in the intestine. The myenteric plexus (Auerbach plexus) lies within the longitudinal and the circular muscle layer, whereas the submucosal plexus (Meissner plexus) is located below the circular muscle layer. The transverse view of the intestinal wall shows the cellular composition of the ENS including neurons and glial cells. The innervation of the ENS is classified as intrinsic, if the neuron's cell body lies within the intestinal wall and extrinsic, if the cell body of the neuron is located outside the intestinal wall.

The term “gut-brain-axis” illustrates the bidirectional communication between the central nervous system (CNS) and the intestine that includes the autonomous nervous and the neuroendocrine system via the hypothalamic-pituitary-adrenal-axis. The autonomous nervous system provides an anatomical cue connecting the CNS with the peripheral tissues (Figure 1). Generally, the innervation of tissues can be classified as intrinsic, if the neuron's cell body lies within the respective tissue and extrinsic, if the cell body of the neuron is located outside the tissue (Figure 2) (12). The ENS belongs together with the sympathetic and parasympathetic nervous system to the autonomous nervous system. Even though the ENS receives input from the CNS, it largely functions independently suggesting a hierarchical structural organization. As a matter of fact, the majority of neurons in the vagal nerve are afferent and thus transmit signals from the intestine to the CNS suggesting that the brain is rather a signal receiver that perceives and integrates signals arising from the gut in order to quickly react to potential danger, damage, or threat (13). The pivotal role of the ENS is highlighted in Hirschsprung's disease, a disorder characterized by congenital lack of enteric neurons. The consequential lack of coordinated propulsive motility pattern in the colon mediated by the ENS results in high morbidity and mortality (14). The crucial role of the ENS for body homeostasis is also illustrated in enteric infections that affect enteric neurons, such as in Chagas disease, which may cause acquired loss of enteric neurons resulting in megaviscera with potential life threatening complications (15).

The ENS is organized in afferent/sensory neurons that transfer information to the CNS, and efferent/motor neurons that transmit signals from the CNS to the periphery. Upon stimulation, somatosensory information is further processed/integrated by dorsal root ganglia located in proximity to the spinal cord. The effector function of the nervous system can be categorized into a somatic and an autonomous arm. The somatic efferent system originates from the brainstem and spinal cord and forms motor neurons that innervate skeletal muscles. The effector function of the somatic neuronal system can be consciously controlled. The autonomous nervous system on the other hand is largely independent of CNS control and can be further subdivided into the sympathetic nervous system, parasympathetic nervous system and ENS (Figure 1, right vs. left panel). The sympathetic and parasympathetic nervous system are anatomically distinct and in many aspects designed to be biochemical and functional counter players (16). The conserved function of sympathetic neurons is to elicit a fight-or-flight reaction, whereas parasympathetic neurons activate a rest-and-digest reaction. The cell bodies of the preganglionic neurons from the sympathetic nervous system are localized in the thoraco-lumbar region, which receive input from the brain stem, hypothalamus and the formation reticularis. Preganglionic sympathetic neurons synapse with postganglionic neuron located in the latero-dorsal thoracolumbar region, which is also referred to as sympathetic trunk. After signal transmission, the long postganglionic sympathetic neurons innervate the gastro-intestinal tract and maintain tissue homeostasis. The cell bodies of parasympathetic preganglionic neurons on the other hand are located in the brainstem and the pelvic sacral nerves. Similar to the sympathetic, the parasympathetic nervous system transmits signals from the brainstem to the respective organs via two neurons. However, the postganglionic parasympathetic neurons are localized in immediate proximity to the target organ. The parasympathetic nervous system innervates the gastro-intestinal tract with nerve fibers from the vagal nerve that end just before the splenic flexure of the transverse colon (also known as Cannon's point) and afterwards with fibers originating from pelvic sacral nerves. Both, the sympathetic and parasympathetic preganglionic neurons are cholinergic and predominantly express and secrete the neurotransmitter acetylcholine (Figure 1; neurotransmitters highlighted in red/green/yellow). The parasympathetic postganglionic neurons are also cholinergic whereas the sympathetic postganglionic sympathetic neurons are catecholaminergic and predominantly express and secrete norepinephrine as a neurotransmitter. The identification of the respective neuronal subsets is therefore based on the expression of enzymes involved in neurotransmitter synthesis such as tyrosine hydroxylase for sympathetic catecholaminergic neurons and choline acetyltransferase (Chat) for parasympathetic cholinergic neurons (17). Because many different immune cells express the receptor for norepinephrine, such as α- and β-adrenoreceptors, the sympathetic nervous system is tightly linked to immune regulation (18). Potentially as a part of the fight-and-flight reaction that need to ensure survival of the organism, the sympathetic nervous system initially has a pro-inflammatory function (19). In long term, the sympathetic nervous system rather suppresses inflammation via β-adrenergic receptors expressed on Neutrophils, Macrophages, innate lymphoid cells (ILCs) and other immune cells (20–25). The exact response of catecholamines, however, is also context-dependent for example on environment and local challenges, co-stimulatory factors and activation levels of cells (19). The parasympathetic nervous system acts via secretion of acetylcholine, which binds to muscarinic and nicotinic acetylcholine receptors. In general, acetylcholine has a rather anti-inflammatory effect following activation. This can be observed upon stimulation of the vagal nerve, which has been termed the “cholinergic anti-inflammatory reflex” (26). Apart from controlling vegetative functions, acetylcholine and norepinephrine regulate cytokine secretion of hematopoietic cells. Vice versa, neurons express cytokine receptors to adequately react on inflammatory stimuli (27). The effect of autonomic innervation of lymphoid organs has been highlighted in the spleen, which is innervated by the superior mesenteric ganglion and eventually the splenic nerve (Figure 1). Especially in the white pulp, T- and B-cell as well as macrophages are in close contact with neuronal innervation (28). Functionally, the interaction of the autonomous nervous system and immune cells control local and systemic inflammation via the cholinergic anti-inflammatory pathway (26). This anti-inflammatory pathway originates anatomically from the vagal nerve that innervates abdominal organs and controls the release of its predominant neurotransmitter acetylcholine. Activation of the vagal nerve lowers the systemic inflammatory response via inhibition of TNF production by myeloid cells (29).

The ENS is organized in plexuses throughout the intestine composed of neurons whose cell bodies lie within the intestinal wall (Figure 2). It forms a continuous modality and ranges from the upper esophagus to the internal anal sphincter. The ENS is the largest accumulation of neurons outside the CNS and contains 100 to 500 million neurons and is thus referred to as the “abdominal brain” (30, 31). The myenteric plexus (Auerbach plexus) lies between the longitudinal and circular muscle layer in the intestinal wall whereas the submucosal plexus (Meissner plexus) forms a network within the submucosal layer (Figure 2). The ENS forms a dense network that mainly includes neurons and glial cells and controls peristalsis, blood flow and maintains water and electrolyte homeostasis. Neurons in the ENS can be categorized based on their anatomy, function and neurotransmitter signature. Up to 20 functional classes of neurons can be identified in the guinea pig. Functionally and phenotypically, several types of enteric neurons are distinguished and can be further sub-classified: Excitatory neurons innervating intestinal muscles, inhibitory neurons innervating intestinal muscles (to circular and longitudinal muscles, respectively), secretomotor and vasodilator neurons, secretomotor neurons without vasodilator activity and neurons to enteroendocrine cells, sensory intrinsic primary afferent neurons, ascending and descending interneurons and intestinofugal neurons (32–34). Single-cell sequencing experiments revealed nine clusters of enteric neurons in mice, which can be classified based on the two neurotransmitters nitric oxide (NO) (Nos1 expression, cluster 1–3) and acetylcholine (Chat expression, cluster 4–9) (35). Structurally, Nos1^+^ neurons are preferentially type I neurons whereas among Chat^+^ neurons type II neurons are overrepresented (34). Additional neurotransmitters include, gamma-Aminobutyric acid (GABA), epinephrine and dopamine, but also vasoactive intestinal peptide (VIP), neuromedin U (NMU), calcitonin gene-related peptide (CGRP), Substance P, Galanin, Tachykinin, and others (32, 35). With regard to neuronal regulation of immune responses, the biochemical signature of the neuron (neurotransmitters, neuropeptides) appear to be functionally most relevant, since many of the neuropeptides such as VIP, NMU, CGRP regulate immune responses via different subsets of immune cells (36–44). The in-depth characterization of enteric neurons may allow to identify neuronal subsets based on the expression of neurotransmitters/neuropeptides and to assign specific inflammatory functions analog to immune cells. However, how the immune modulatory function of neuronal factors is linked to their physiological function is still poorly understood. Such insights would provide an integrated view on the regulation of intestinal and immune homeostasis. For example, it is well-established that inhibitory motor neurons in the ENS are characterized by co-expression of NO and VIP as main neurotransmitters (34, 35). However, how the inhibition of motor activity is linked to regulation of immune cells and what are the respective stimuli for their release remains poorly understood.

## Neuronal Afferent Signals Modulate Tissue Immunity

Sensory neurons play an important role in detecting harmful environmental challenges, transmit these signals to the CNS and allow for an adequate reaction against potential pathogenic threats or tissue damage. Recent evidence suggests that the CNS receives direct neuronal afferent signals upon the input from gut enteroendocrine sensory cells. Enteroendocrine cells are gut epithelial cells that form a tight connection with vagal neurons. This interaction builds the basis of a neuro-epithelial circuit to the CNS that senses gut stimuli via glutamate as the main neurotransmitter (45). However, this finding is controversial because another study did not observe direct neuronal contact with epithelial cells (46). Thus, further experiments need to clarify the exact interaction between sensory neurons and epithelial cells. Sensory neurons respond to a broad variety of chemical and physical stimuli that can activate different ion channels, such as transient receptor potential vanilloid (TRPV1), transient receptor potential ankyrin 1 (TRPA1) and transient receptor potential cation channel subfamily M member 8 (TRPM8) (18, 47). An important class of sensory neurons are nociceptors that are able to detect noxious stimuli such as heat, chemical and mechanical perturbations (48). The role of nociceptors in sensing a broad variety of stimuli, and in turn, regulating immunological functions has been proposed by several studies (49–54). For example, sensing of type 2 cytokines, such as Interleukin (IL)-4, IL-5, IL-13 directly activate sensory neurons and promote chronic itch that is dependent on neuronal IL-4Rα and JAK1 signaling (53). Interestingly, JAK inhibitors improved chronic itch in patients, even after failure of state-of-the-art immunosuppressive therapy and therefore represent a novel treatment option for atopic dermatitis (53). In addition to type 2 effector cytokines, the epithelial cell-derived alarmin, thymic stromal lymphopoetin (TSLP), which is an important initiator of type 2 immune responses, can activate TRPA1^+^ sensory neurons in the skin and induce itch behavior in mice (50). Besides itch, also pain-sensitizations have been proposed to be induced by bacterial products following direct activation of nociceptor sensory neurons (49, 55). In fact, Nav1.8^+^ neurons sense bacteria-derived N-formylated peptides and α-hemolysin suggesting that pain can be a direct consequence of neuronal sensing of bacteria during certain infections in addition to the reaction to immune activation or inflammation (49). Meseguer and colleagues found that lipopolysaccharide (LPS) was able to directly stimulate excitatory actions on TRPA1^+^ neurons and thus eliciting nociceptor activity and eventually pain (55). The finding that bacteria directly induce pain-sensitizations is intriguing because subclinical, not overt low grade infections may be causative for different chronic pain syndromes in humans. Thus, blocking of specific, bacteria-derived neuronal sensitizations may be a valuable treatment option for such chronic pain syndromes (56). These studies further unraveled the potential of pathogen-sensing via the autonomous nervous system that has classically been attributed to pathogen-receptors expressed on immune cells. Therefore, the autonomous nervous system may be an important player in the establishment of host-microbial mutualism. Another fact that has not yet been deeply addressed is the expression of classical pattern-recognition receptors, such as toll-like receptors (TLRs) 2, 4, and 7 on enteric neurons, which have been well-studied on myeloid and epithelial cells (57, 58). If we consider the broad variety of existing TLR-ligands, the ENS may therefore be an unprecedented player in pathogen recognition. In fact, the ENS has recently been shown to directly recognize parasite-derived excretory-secretory products in a Myd88-dependent fashion during *Nippostrongylus brasiliensis* (*N. brasiliensis*) infection in mice underlining the concept of pathogen-sensing by the ENS (37). Furthermore, viruses can stimulate different TLRs, thus broadening the functional role of the ENS in mounting immune reactions against infections (59, 60). However, there is a fundamental lack in knowledge of how the ENS can sense these signals and consequently adapt immune effector functions. While functional studies investigating the role of TLRs in neurons are scarce, several reports highlight the importance of TLRs in glial cells. Deletion of the signaling adapter molecule MyD88 on glial cells, which transduces signals of many TLRs but also IL-1 cytokine receptors such as IL-1R, IL-33R, IL-18R, resulted in decreased ILC activation during DSS colitis and *N. brasiliensis* infection and suggests that there is TLR-mediated sensing of pathogens by the ENS and vice versa leading to immune activation (37, 61). Especially TLR2 seems to play an important role in controlling ENS architecture and consequently intestinal inflammation via glial cell-derived neurotrophic factor (GDNF) (62). In fact, enteric neurons from TLR2^−/−^ mice had smaller ganglia, fewer HuC/D^+^ and nNOS^+^ neurons as wells as shorter betaIII-tubulin axonal networks, whereas supplementation with GDNF corrected the observed phenotype (62). Nociceptors on the other hand release neuropeptides, such as CGRP, substance P, and VIP, which can adapt the local immune function depending on the milieu and the local challenge. That neurons have the ability to directly sense and integrate signals may also be represented by the fact that the microbial colonization has an important impact on neurophysiology and behavior (63, 64). In fact, germ-free mice, which are devoid of any microbial exposure, develop relevant alterations in behavior and the microbiota seem to be relevant to different neurodegenerative diseases (65, 66). A detailed discussion of the gut-brain axis is, however, beyond the scope of this review and the reader is kindly referred to excellent articles (64, 67).

## The Enteric Nervous System Integrates Signals From Commensal Microbiota

The microbiota inhabit all mammalian body surfaces and play a pivotal role in the education of the host immune system (68). Recent evidence now suggests that the presence of commensal microbiota also shape the neuronal gene programs and eventually the extrinsic sympathetic activity (46, 69). Muller and colleagues proposed that the commensal microbiota shape intrinsic enteric-associated neuronal programs (EAN) region-dependent along the intestine, whereas intrinsic EAN are functionally adapted to the specific intestinal region and its associated microbial challenge. Interestingly, germ-free mice exhibited hyperactivation of sympathetic neurons whereas the microbial product, butyrate, suppressed sympathetic hyperactivation (46). These results reveal that a metabolite-mediated gut-brain circuit adapt autonomic nervous functions dependent on the local milieu. Because the sympathetic nervous system controls different autonomic nervous functions (blood pressure, heart rate and other) one may speculate that certain human diseases may be caused by alterations in the intestinal microbial composition. Furthermore, the aryl hydrocarbon receptor (*AhR*) has been shown to be expressed by virtually all myenteric neurons in the colon and the distal small intestine in specific-pathogen-free (SPF)-colonized mice whereas its expression was absent in the duodenum and jejunum of SPF-colonized mice or in the colon of germ-free mice suggesting that the microbial colonization dictates *AhR* expression. Enteric neuron-specific deletion of *AhR* resulted in an increase in gut-transit time whereas supplementation of the Ahr-ligand, I3C, restored intestinal transit time suggesting that neuron-specific and ligand-dependent activation of *AhR* controls intestinal motility (69). Taken together, these studies reveal a link between the intestinal microbiota and enteric neurons suggesting that enteric neurons constantly sense the commensal microbiota in order to maintaining body homeostasis.

## Cellular Mechanistics of Neuro-immune Interactions

### Innate Lymphoid Cells (ILCs)

ILCs enclose diverse populations of innate immune cells, which are derived from the common lymphoid precursors, but which lack rearranged antigen-specific receptors and thus develop independently of Rag recombination (70, 71). Based on developmental and functional aspects, two different main groups of ILCs are distinguished, cytotoxic ILCs [conventional natural killer (NK) cells] and helper-like ILCs (ILC 1, 2, and 3) (72, 73). Conventional natural killer cells (NK cells) are known since the 1970s because they are well-represented in the blood and secondary lymphoid organs (74, 75). NK cells are developmentally dependent on the transcription factor Eomesodermin and mediate immunity to intracellular pathogens and tumors. The immunology of helper-like ILCs was mainly studied in the last 10 years (76) with the exception of lymphoid organ development mediated by lymphoid tissue inducer cells (LTi cells) (77). The reason for the late discovery has been discussed (78) but one reason might be the enrichment of helper-like ILCs at barrier surfaces, which were less in research focus at that time. Helper-like ILCs are characterized based on the expression of and developmentally dependency on lineage-specifying transcription factors and the effector cytokine profile: (i) ILC1s require T-bet and secrete IFN-γ and TNF and are involved in control of mainly intracellular pathogens, (ii) ILC2s require GATA-3 and BCL11b and secrete IL-5, IL-9, and IL-13 to combat helminth infections or to drive allergic reactions, and (iii) ILC3s are RORγt dependent IL-22 secreting cells, which maintain barrier integrity and protect from intestinal infections. ILC3s can be subdivided in CCR6^+^ LTi-like producing IL-17A and CCR6^−^ ILC3, which co-express T-bet and IFN-γ and have the potential to differentiate into ILC1-like cells. For a more detailed overview on ILCs biology the reader is kindly referred to more comprehensive reviews on this is topic (76, 79, 80).

ILCs are mainly located at barrier surfaces and act as a first line of defense against potentially invading microbes and are establishing host-microbial interactions. In addition to host-defense mechanisms, ILCs have also been implicated to contribute to tissue repair and maintenance of barrier integrity and organ homeostasis (81–84). In order to fulfill this function, ILCs need to be rapidly activated. However, in contrast to myeloid cells, the expression of pattern recognition receptors is very limited in ILCs suggesting that they do not sense danger signals directly by expression of pattern recognition receptors. Instead, they are activated indirectly by cytokines secreted by other cells in tissues, e.g. by alarmins and other cytokines such as IL-12 and IL-15 for ILC1s, IL-25, IL-33, and TSLP for ILC2s, IL-1β, IL-23, and TL1A for ILC3s (79, 85, 86).

**Figure 3:**
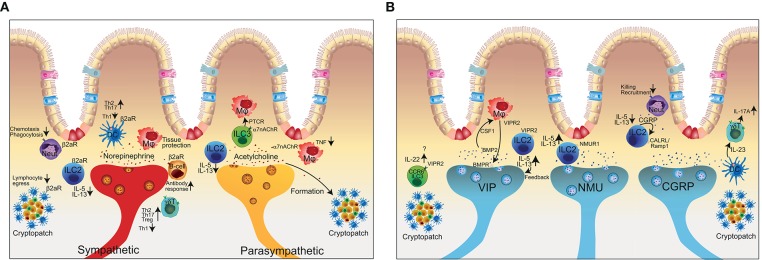
The complex interactions of the enteric nervous system with innate and adaptive immune cells. **(A)** Representation of the close co-localization of extrinsic neuronal fibers and immune cells in the intestine. Sympathetic signal transmission (red axon) using (nor-)epinephrine can exert pro- and anti-inflammatory effects depending on the immune cell and respective receptor activation. The sympathetic nervous system in addition controls lymphocyte egress from lymph nodes. The parasympathetic nervous system (yellow axon) in general has anti-inflammatory effects but is also involved in the formation of tertiary lymphoid organs. **(B)** Schematic representation of intrinsic neuronal subsets of the enteric nervous system (VIP, NMU, CGRP) and the interaction with immune cells. Neut, neutrophil granulocyte; DC, dendritic cell; Mϕ, macrophage; ILC, innate lymphoid cell; γδT, gamma-delta T cell; α7nAChR, alpha7 nicotinic acetylcholine receptor; β2aR, beta2 adrenergic receptor; VIP, vasoactive intestinal peptide; NMU, neuromedin U; CGRP, calcitonin-gene related peptide; VIPR2, vasoactive intestinal peptide receptor 2; NMUR1, Neuronmedin U receptor 1.

Since ILCs are present in large numbers in the intestine, which is also densely innervated by the ENS and the other components of the autonomic nervous system, neuronal factors emerged as potential regulators of immune responses and sensors for danger signals. Indeed, recent research has provided evidence that ILCs integrate neuronal signals and express receptors for neuropeptides and neurotransmitters (Figure 3) (23, 36–42, 44, 87, 88). ILC responses are regulated by the ENS as well as the sympathetic and parasympathetic nervous system, glial cells and by endocrine loops (89).

The sympathetic arm and the respective neurotransmitter norepinephrine has been shown to inhibit ILC2s and consequently decrease type 2 responses via β2-adrenergic receptors (Figure 3A). Absence of the β2-adrenergic receptor on ILC2s in a mouse model of *N. brasiliensis* magnified the type 2 immune reaction and resulted in improved worm clearance (23). Similar to the cholinergic anti-inflammatory pathway, regulation of acetylcholine on ILC2s has been shown to bind on alpha7-nicotinic acetylcholine receptors (α7nAChR) (Figure 3A). Administration of a specific agonist for α7nAChR on ILC2s reduced ILC2 effector function and eventually dampened allergic lung inflammation (88). In contrast, vagotomy and lack of acetylcholine results in a delayed resolution of Escherichia coli infection via peritoneal ILC3 (90). Mechanistically, abrogation of vagal neuropeptides functionally decreased secretion of the immunoresolvent PCTR1 by peritoneal ILC3 whereas supplementation of either PCTR1 or ILC3 restored host responses against *E.coli*. These results suggest that the cholinergic modulation has a tissue protective role and shapes the ILC3 compartment to regulate tissue homeostasis (91). Furthermore, signals from the vagal nerve regulate the formation of tertiary lymphoid tissue during chronic inflammation (92). However, how much LTi cells are involved as receivers of neuronal signals in this process requires further investigation.

ILC3 are critical in host-defense at mucosal sites and regulators in inflammation. Recent data show that adult CCR6^+^ ILC3 express the neurotrophic receptor RET and ILC3-autonomous RET ablation decreased IL-22 production and increased the susceptibility to bowel inflammation and infection suggesting a modulatory interaction of the nervous system with ILC3 (61). Microbial sensing of the microenvironment is mediated by glial cells adjacent to cryptopatches, in which the CCR6^+^ ILC3s are located. Upon sensing of microbial-associated molecular patterns, glial cells released the RET-ligand glial cell-derived neurotrophic factor (GDNF) to stimulate IL-22 production of CCR6^+^ ILC3s in the cryptopatch. (61). Within intestinal tissues, ILCs and nerves show a close co-localization, which presumably supports neuronal regulation of ILC responses. Enteric neurons express the neuropeptides NMU, VIP, and CGRP whereas the receptors for these neuropeptides are expressed on ILCs (Figure 3B) (37–44).

NMU is a highly conserved neuropeptide, which is generated by proteolytic cleavage of a pro-protein by unknown proteases into bioactive small peptide fragments (93). NMU is mainly expressed in the thalamus in the CNS and enteric neurons within the gastrointestinal tract. NMU binds to two large G-protein coupled receptors, coined NMUR1 and NMUR2. While NMUR2 is mainly expressed by neurons, NMUR1 was found to be selectively expressed by ILC2s (Figure 3B) (37–39, 90, 93). Furthermore, NMU was a very strong stimulator of ILC2s and triggered type 2 immune responses promoting anti-helminth immunity in the intestine or in the context of lung inflammation via NMUR1 (37, 38). Interestingly, NMU was shown to be upregulated during helminth infection and enteric neurons were shown to directly sense worm-derived excretory/secretory products in a Myd88-dependent manner and react to that stimuli with the production of NMU as an immune effector molecule. Altogether these data support a model where cholinergic neurons regulate type 2 inflammation via production of NMU and engagement of NMUR1 on ILC2s.

While NMU from cholinergic neurons stimulate ILC2s, the neuropeptide CGRP is also secreted by neurons with a cholinergic signature but in contrast inhibits ILC2 activation (Figure 3B). Interestingly, ILC2 also produce CGRP themselves in addition to being equipped with the receptors for CGRP CALCRL/Ramp1. Therefore, CGRP might act as a negative feedback loop to control ILC2 activation. CALCRL/Ramp1 engagement by CGRP binding triggers a signaling cascade in ILC2s, which signals via Gαs proteins and regulate intracellular cAMP levels. ILC2 activation is suppressed by CGRP and genetic deletion of components of the CGRP—CALCRL pathway resulted in elevated ILC2s responsiveness and type 2 inflammation in the context of helminth infection, lung inflammation and food allergy (40–43). Other findings uncovered the regulation of ILC2s by neuroendocrine cells (94) and tuft cells that share many commonalities with neurons (Chat-expression, sensing, signal transmission). Pulmonary neuroendocrine cells on one hand secrete CGRP and GABA and reside in close proximity to ILC2 in the lung. In models of allergic asthma, neuroendocrine cells are pivotal players in regulating ILC2s and consequently mucosal type 2 responses (94). Tuft cells on the other hand are chemosensory cells in the intestine and the major source of IL-25 and can activate ILC2 to mount type 2 responses (95–97). The commonalities of these cell types with the nervous system however requires further investigation.

While Nmur1 was reported to be selectively expressed by ILC2, Vipr2, one receptor for VIP, is expressed on both ILC2 and ILC3 (Figure 3B). VIP, known as the circadian synchronizer, has been shown to stimulate ILC2 via Vipr2 resulting in the release of IL-5. (87). Nav1.8^+^ nociceptors in the lung secrete VIP upon stimulation and the resulting induction of IL-5 has been linked to eosinophil accumulation and consequently worsened ovalbumin-induced lung inflammation (87). The ILC2-mediated production of IL-5 further increased the nociceptor stimulation in the sense of a backward loop (36). VIP can also adapt ILC3 function in the intestine dependent on the day and oscillating between active and resting phases (41). ILC3 expressed high levels of *Vipr2* whereas VIP induced the IL-22 production that has been shown to be an important player in maintaining bowel integrity (98). In fact, genetic deletion of the VIP-Vipr2 pathway by using *Vipr2*^−/−^ mice resulted in an increased susceptibility to DSS-colitis (41). A study by Talbot and colleagues found that CCR6^+^ ILC3s but not CCR6^−^ ILC3s, in cryptopatches expressed Vipr2 and additional molecules related to neuro-immune interaction. CCR6^+^ ILC3s are an important source of IL-22, which regulates epithelial function including production of antimicrobial peptides and lipid absorption and thereby adapting the immune control to nutrient uptake (44). While the VIP–VIPR2 pathway links antimicrobial immunity, circadian rhythm and food adsorption, whether ILC3 are stimulated or inhibited by VIP is controversial. Seillet and colleagues measured that VIP induces ILC3s and IL-22 production whereas Talbot et al. found inhibition of ILC3s and IL-22 by VIP (41, 44). Therefore, further experiments need to be conducted to investigate context-dependent effects of VIP.

The hypothalamic-pituitary-adrenal axis (HPA) regulates the immune system via control of glucocorticoid secretion, which is key negative regulator of hematopoetic cells. Although secreted by the adrenal gland, the production of glucocorticoids is under control of the CNS and, therefore, linked to neuronal regulation of immune responses. Quatrini and colleagus recently showed that the regulation of natural killer (NK) cell function is dependent on the glucocorticoid receptor (GR) for resistance to sepsis and for immunopathology in the context of murine cytomegaly virus infection. Mechanistically, endogenous glucocorticoids induced the expression of PD-1 on NK cells and limited the production of IFN-ɤ eventually preventing mortality in infected mice (99, 100). These results highlight the importance of further studies that investigate the functional role of the HPA axis in tuning or downregulating immune functions.

In summary, neuronal regulation of ILCs via multiple neuropeptides and neurotransmitters and the corresponding receptors expressed by ILCs, emerged as an important signaling hub in tissues for integration of body homeostasis and immunity at barrier surfaces.

### Dendritic Cells

Dendritic cells (DC) are classical antigen-presenting cells that express pattern recognition receptor (PRRs) to sense the environment for the presence of danger signals and if necessary initiate an immune response against the pathogenic encounter. In addition to PRRs, DCs express adrenergic receptors and receptors for neuropeptides suggesting a modulative effect of the autonomous nervous system in mounting immune responses (22). In fact, β2-adrenergic stimulation of DCs results in skewing the T cell response toward Th2 and Th17 responses at the costs of Th1 promotion (Figure 3A) (22). However, conclusions drawn from these findings are limited because experiments, which demonstrate the importance of DC-neuron interaction via β2-adrenergic receptors *in vivo* are still missing (25).

The interaction of neurons and DCs has recently been highlighted in the skin in the context of Candida albicans (*C. albicans*) infections and Psoriasis-like inflammation (51, 52). Nociceptive signals by *C. albicans* in the skin can directly induce the secretion of CGRP. Such stimuli lead to the production of IL-23 by DCs further resulting in activation of ɤδT cells and secretion of IL-17A (Figure 3B). Notably, the absence of sensory neurons increased the susceptibility to *C. albicans* infections suggesting that neurons sense pathogens in order to control infections in close interaction with DCs (52). Another study showed that DCs are in close contact to nociceptive neurons and express the ion channels TRPV1 and Nav1.8 in the skin. Ablation of nociceptors led to failure of IL-23 production by DCs and consequently did not induce inflammatory cytokine production by ɤδT cells. Interruption of this neuro-immune cue failed to recruit inflammatory cells upon infection suggesting that TRPV1^+^ Nav1.8^+^ nociceptors regulate the IL-23/IL-17 pathway and control cutaneous immune responses (51). These experiments suggest a clear link between the neuro-DC interaction and skin disease pathogenesis. However, the expression of a broad variety of receptors for neuropeptides/neurotransmitters on DCs remains a blackbox and studies need to delineate the role of the DC-neuron interaction in steady-state and other disease models.

### Neutrophils

Neutrophils express and release a large variety of cytokines to regulate inflammatory reactions, and to recruit and activate other cells of the immune system. In addition, they have the ability for engulfment and intracellular killing and thus are players at the front-line of defense against invading pathogens (101). Because these cells act at the fore-front of tissue damage, the neuronal signaling may be obvious because of the urgency of infections and the need for cell recruitment.

In the context of Streptococcus pyogenes infection in the skin, bacteria can directly activate nociceptive neurons via secretion of streptolysin S. Activation of nociceptors resulted in the release of the neuropeptide CGRP that inhibited the recruitment of neutrophils and phagocytic killing that can be seen as a hide-me signal of bacteria (Figure 3B) (56). Interestingly, Botulinum neurotoxin A and CGRP antagonists reversed the suppressed immune-reaction suggesting that this may be a valuable strategy to overcome the pathogenicity of highly invasive bacterial infections. In line with this data and in a model of Staphylococcus aureus pneumonia, TRPV1^+^ nociceptors suppressed the recruitment of neutrophils and altered ɤδT cells whereas this inflammatory suppression worsened survival, cytokine production and bacterial clearance (54). Another study that highlights the role of neuronal-neutrophil crosstalk has shown that noradrenalin suppressed chemotaxis and phagocytosis in a stroke model (Figure 3A) (24).

Taken together, there is good evidence that neuronal sensing of microbes shapes immune responses at barrier surfaces such as the skin and the lung. However, there is a fundamental lack of knowledge on the complex interaction of neurotransmitters and neuropeptides at other barrier surfaces such as the intestine. For example, because the receptor for CGRP CALCRL/Ramp1 is relatively broadly expressed on immune cells, future studies need to address if the adaptation of neutrophil function is a rather direct effect mounted by CGRP itself or an indirect effect through functional changes of other cells. A direct effect of neurons on neutrophil function would be intriguing because it could explain rapid cell recruitment during inflammation. However, further studies need to address the expression and function of specific neuropeptide or neurotransmitter receptors on neutrophils.

### Macrophages

Macrophages are specialized phagocytes that are located in most body tissues. As a part of the innate immune system, macrophages help keeping the organism clean and restore tissue damage. Thus, they process dead cells, debris, foreign bodies, and initiate inflammatory processes via antigen-presentation. The expression pattern of receptors for neuropeptides and neurotransmitters on macrophages suggests that neurons and macrophages are closely linked in order to regulate tissue homeostasis and to fight infections.

The pivotal role of macrophages in integrating cholinergic signals resulting in a profound anti-inflammatory effect has been shown by the group of Tracey, which has been termed the “cholinergic anti-inflammatory pathway” (Figure 3A) (102). The initial observation that vagus nerve stimulation prevented the development of septic shock in mice implies neuronal control of macrophage function in acute disease (11). Later on, the group of Tracey discovered that vagal signals are transmitted via acetylcholine that binds α7 nicotinic acetylcholine receptors (α7nAChR) expressed on macrophages and results in dampening of TNF production (102). If we consider the speed of neuronal conductance, central stimuli are capable of instantaneous cell recruitment and modulatory signals to the site of inflammation (8). Another example of cholinergic vagal control of inflammation via macrophages has been shown in a model of postoperative ileus. The α7nAChR was expressed on muscularis macrophages and controlled postoperative ileus formation whereas stimulation of the vagal nerve attenuated surgery-induced intestinal inflammation (103). The interplay and the tight connection of macrophages located within the longitudinal and circular muscle layer in close contact with the myenteric plexus (Figure 2) not only controls postoperative ileus formation, but is also involved in the pathogenesis of diabetic-induced gastroparesis (104). There are clear parallels between the autonomous nervous-macrophage interaction in the periphery and the interaction of tissue-resident macrophages within the CNS (105). This close proximity of biologic functions in different tissues has been suggested because neuronal signals in the CNS keep tissue-resident macrophages at a quiescent state and macrophages in the CNS express high levels of CX3CR1, a pattern that has been postulated to be unique for tissue resident-macrophages in the CNS, the microglia (105).

In the intestine, the growth factor for macrophage development, colony stimulatory factor 1 (CSF1), is secreted by the nervous system and controls gastrointestinal motility. Reciprocally, macrophages sense the microbiota and change the pattern of smooth muscle contractions via bone morphogenic protein 2 (BMP2) binding on the BMP receptor expressed on enteric neurons (Figure 3B) (106). These results suggest a reciprocal tight regulation of gastrointestinal motility via the interaction of muscularis macrophages and enteric neurons that in turn depend on signal input from the intestinal microbiota.

The anatomic location of intestinal macrophages is highly specialized dependent on the proximity to the gut-lumen. In fact, lamina propria macrophages represent a rather pro-inflammatory phenotype in comparison to macrophages located in the muscularis that represent a rather tissue-protective phenotype. Extrinsic sympathetic neurons mediate tissue-protective effects via activation of β2-adrenergic receptors expressed on macrophages in the muscular sheet (Figure 3A) (21). Furthermore, intestinal muscularis macrophages protect neurons from cell-death via β2-adrenergic mediated upregulation of neuroprotective programs (107). Taken together, macrophage function is highly dependent on the signal input from the autonomous nervous system and vice versa to rapidly react to infectious stimuli and tissue damage.

### Mast Cells

Urticaria, a common psycho-dermatological disorder, is the result of vascular dilation, edema, and the immediate release of histamine by mast cells in the skin (108). It has been suggested that psychological stress is strongly involved in the pathogenesis of urticaria underlining the role of neuronal triggers to effector cells such as mast cells (109). Throughout the gastrointestinal tract, mast cells are located in close proximity to sensory nerve fibers (110). Mast cells contain granules rich in histamine and heparin, which can be immediately released and trigger rapid responses such as allergic reactions or anaphylaxis.

Mast cells have also been implicated in atopic dermatitis, where dermal lesions are hyper-innervated with a high abundance of substance P fibers and an increased respective receptor expression on mast cells (111). Nerve-derived substance P induced the rapid release of histamine, TNF, leukotriene B4, and vascular endothelial growth factor by mast cells suggesting a close interaction of neurons and mast cells in allergic diseases (18, 112, 113). This neuronal-mast cell connection has been underlined in the context of allergic skin disease models in mice. House dust mites directly activated TRPV1^+^ nociceptive sensory neurons driving the development of allergic skin inflammation via the secretion of substance P that eventually resulted in degranulation of mast cells (113). This data provides an important signaling pathway that may be the mechanistical basis for a broad variety of allergic diseases. Psychological stress additionally triggers the release of neuropeptides (Substance P, Corticotropin Releasing Hormone) that act on mast cells and promote the release of mast cell mediators (114). Interestingly, mast cells but not eosinophils or T-cells were associated with asthmatic diseases in patients underlining the importance of these cells for allergy development in humans (115).

### T- and B-Cells

Autonomous nervous fibers innervate lymphoid organs such as mesenteric lymph nodes and Peyer's patches (116, 117). There is evidence that lymph nodes may receive neural afferent innervation in addition to the sympathetic efferent innervation that may suggest neuronal sensing of imminent immunologic threats whereas such coordinated actions direct the immune system to sites of injury and infection (118, 119). The close proximity to adaptive immune cells suggests that nerve fibers participate in neuro-immune cross-talk and modulate signals from the adaptive immune system. Sympathetic neurotransmitters such as epinephrine and norepinephrine predominantly bind β2-adrenergic receptors that are highly expressed on B cells and to a lower level in CD4^+^ T cells (Figure 3A) (20). Activation of β2-adrenergic receptors in general increase intracellular cAMP that activates protein kinase A. Such activation of the B-cell compartment via β2-adrenergic receptors seems to be needed for maintenance of an optimal antibody response suggesting that the autonomous nervous system controls and shapes the magnitude of immune responses (120). In line with the effects observed in B-cells, T cells and the release of their effector cytokines are controlled via sympathetic activity whereas sympathetic innervation suppresses Th1 and promotes Th2, Th17 and Treg responses (20, 25). Another finding that supports the notion that the autonomous nervous system controls adaptive immune functions and recruits cells to effector sites is that activation of β2-adrenergic receptors enhanced retention-promoting signals and inhibited lymphocyte egress from lymph nodes (121). Such migratory effects are dependent on circadian regulation in the T-cell compartment suggesting that the magnitude of adaptive immune responses can depend on neuronal-regulated signaling input from the CNS (122). It should be noted that the β2-adrenergic receptor was reported to control ILC2 and macrophage activation. Thus, further experiments need to clarify if the modulation of T-cell function is rather a consequence of the release of cytokines by other cells or delineate the exact downstream effects upon β2-adrenergic receptor activation in steady-state and disease.

## Neuro-immune Interactions in Disease

### Inflammatory Bowel Disease

**Figure 4:**
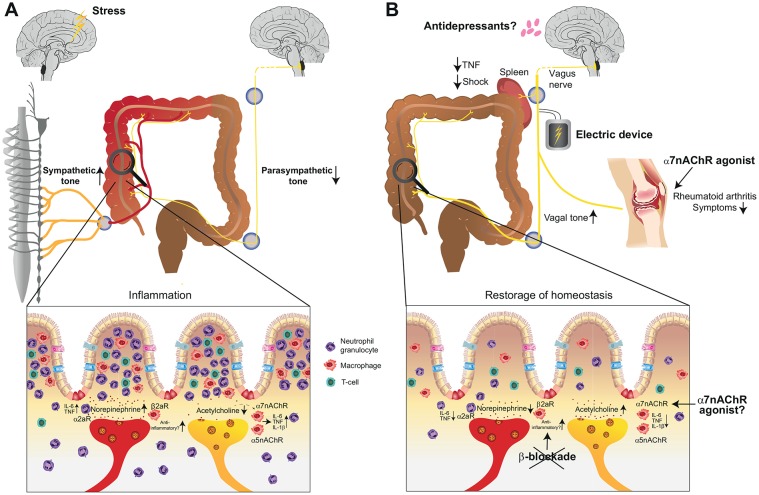
Involvement of the autonomous nervous system in the pathogenesis of inflammatory bowel disease (IBD) and available treatment modalities that harness neuro-immune interactions. **(A)** Stress and the transmitted signals may enhance the sympathetic tone and lead to an additional pro-inflammatory reaction in the intestine that may be causal or at least worsening the course of IBD. Failure to maintain an adequate parasympathetic tone can further support the pro-inflammatory reaction. **(B)** Available treatment options in chronic inflammatory conditions include electric stimulation of the vagal nerve in rheumatoid arthritis, IBD and endotoxin-induced septic shock. Furthermore, the repertoire of treatment strategies for IBD and rheumatoid arthritis may be extended with α7nAChR-agonists. β-blockade should, however, be omitted in IBD. α7nAChR, alpha7 nicotinic acetylcholine receptor; α5nAChR, alpha5 nicotinic acetylcholine receptor; α2aR, alpha2 receptor; β2aR, beta-2 adrenergic receptor; TNF, tumor necrosis factor alpha.

Psychologic disorders show a lifetime prevalence of up to 30% in the general population and major depression may become the most important disease in Western societies (123, 124). In line with the constant increase of psychologic disorders, the incidence of IBD increase as well emerging an unprecedented link between a potential nervous dysregulation and overwhelming immune activation (125). In fact, many patients with IBD have alexithymia that is characterized by the impossibility to verbalize emotions. Such endogenous stress may interfere with body homeostasis and lead to a distorted integrity of the neuro-immune axis that may be causative or at least worsen the clinical course of IBD (126). In mouse models, catecholamines acting on α2-adrenoreceptors led to pro-inflammatory cytokine production worsening dextran sodium sulfate (DSS) colitis. Paradoxically, sympathetic denervation induced clinical signs of colitis (Figure 4A) (127, 128). *In vitro* experiments have shown that norepinephrine blocks the secretion of a variety of proinflammatory cytokines and mice lacking the beta-2-adrenergic receptor were more susceptible to DSS-colitis (129). These studies reveal that sympathetic innervation can have pro- and anti-inflammatory effects and studies need to further clarify its role in IBD. In fact, a retrospective study in humans by using pharmacological inhibition of β-adrenergic receptors showed higher risk for IBD relapse suggesting that solely blocking the pro-inflammatory effect of sympathetic activation may have rather pro-inflammatory effects in long term (130). The parasympathetic tone via the vagal nerve also impedes with IBD. Studies in vagotomised mice showed increased susceptibility to develop colitis upon DSS treatment similar to the anti-inflammatory reflex observed in models of septic shock (131, 132). The absence of the vagal tone was associated with an increase in pro-inflammatory cytokines such as IL-1β, IL-6, and TNF (Figure 4A). These cholinergic signals seem to be transmitted via α7nAChR (131). Apart from the α7nACh receptor, also α5nAChR knockout mice had more severe colitis suggesting that vagal innervation acts in different acetylcholine receptor subunits and modulates immune functions (133). As mentioned above, the psychological distress profile of IBD patients focused the interest on the finding that mucosal levels of acetylcholine in a murine model of depression were associated with more severe colitis in response to DSS suggesting that chronic modulation of the vagal tone enhances the susceptibility to IBD (132). Interestingly, adoptive transfer of macrophages from depressive mice induced inflammatory markers and increased the severity of DSS colitis. These data identified the pivotal role of macrophage in linking stress and susceptibility to intestinal inflammation whereas this effect was reversible with antidepressants (Figures 4A,B) (134). Apart from the importance of macrophages as effector cells of the neuro-immune axis, transfer of CD4^+^ T cells isolated from vagotomised animals resulted in an increased susceptibility to DSS colitis suggesting that more players are involved in cholinergic signal transmission underlining the need to study this interaction in more detail (135). Taken together, the sympathetic and parasympathetic nervous system play important roles in mounting pro- and anti-inflammatory immune reactions in the context of IBD. As a potential therapeutic target in a preclinical model of colitis, the α7nAChR agonist anabaseine, showed considerable effect and the mice developed less weight loss and less severe colitis in a DSS colitis model (Figure 4B) (136). Other reports showed opposite results. Although α7nAChR agonists reduced NF-κB transcriptional activity, IL-6 and TNF release, α7nAChR agonists worsened the effects of DSS-induced colitis or were ineffective in a model of TNBS-induced colitis (137). It is in addition of importance to emphasize that anti-inflammatory effects may lead to an increased susceptibility to infectious diseases (8). Following bacterial peritonitis, virtually all α7nAChR knock-out mice cleared the infection from their peritoneal cavities and had sterile blood cultures mediated via neutrophil recruitment, whereas wild type mice had high bacterial loads at the primary site of infection and were bacteremic (138). These data underline the potential importance of the α7nAChR in host defense. In line with this observations, acetyl-cholinergic agonists, such as nicotine, worsened bacterial clearance and survival upon abdominal sepsis (139, 140). Thus, translation into the clinical setting has to be obtained with caution because solely dampening effector immune function and consequently immune suppression may lead to serious infectious complications. Another interesting approach for the treatment of IBD is the direct electric stimulation of the vagal nerve via an implantable device targeting the anti-inflammatory pathway (Figure 4B). First results have shown improvement in disease activity and endoscopic indices in patients following electric stimulation of the vagal-nerve (141). These results are promising because treatment failure of available biologics is not uncommon and effective treatment is associated with considerable side-effects and mortality in the long term (142). A deeper understanding is needed and may help to uncover novel therapeutic measures for treating IBD. Of note, depression and other psychological disorders may enhance the disease severity and either antidepressive medication or psychological co-therapy may adjust immune functions and lower the severity of the clinical course.

Furthermore, it is of importance to note that enteric glia cells, specialized macrophages in close proximity to neurons, outnumber neurons by 4- to 10-fold (Figure 2) (143). The pivotal role of glial cells in neuro-immune interactions was observed after ablation of enteric glia cells that led to fulminant jejuno-ileitis in mice (144). Enteric glial cells express a broad pattern of neurotransmitters and thereby protect neurons and regulate their activity (145). Enteric glial cells show abnormal behavior in IBD in humans but their role in its pathophysiology has to be further clarified.

### Ileus

Postoperative Ileus is a serious concern in the surgical setting because patients fail to rapidly recover from an operative intervention and remain with symptoms such as nausea, vomiting, and constipation. Following a surgical procedure, postoperative ileus formation is characterized by an over-activation of inhibitory neuronal pathways that triggers inflammation beyond the distant untouched areas and leads to generalized impairment of gastrointestinal motility (146). In fact, low-grade inflammation due to macrophages residing in the intestinal muscularis is key in the induction of postoperative and endotoxin-induced ileus formation (147, 148). Activation of these macrophages mediated the influx of leucocytes at 3–4 days after surgery whereas the inflammatory response impaired normal propulsive neuromuscular function and consequently digestion (146). There is an urgent need to uncover novel pharmacologic targets in the early event of microscopic inflammation that may help to reduce ileus formation. Studies show that ileus onset can be reduced by modulating the cholinergic anti-inflammatory tone (149–151). Interestingly, vagal stimulation reduced surgery-induced inflammation and ameliorated postoperative ileus formation in a STAT3 dependent manner mediated by intestinal macrophages (149). Supportive literature showed that modulation of cholinergic neurons via α7nAChR agonists improved gastrointestinal transit time through inhibition of low-grade inflammation on the basis of macrophages (150, 151).

### Sepsis

The cholinergic anti-inflammatory function via dampening of TNF synthesis has been shown in LPS-induced endotoxemia, whereas stimulation of the vagus nerve protected from the development of shock (11) (Figure 4B). Interestingly, splenectomy abolished the anti-inflammatory effect of the vagal nerve suggesting a pivotal role of the spleen in inflammatory reactions. This observation may explain why the organism is prone to the often fatal overwhelming post-splenectomy syndrome (OPSI) that may serve as an alternative hypothesis to the current thinking that OPSI is a result of impaired clearance of encapsulated bacteria (152). Advances in the mechanistic understanding of this observed phenotype exposed that acetylcholine signals via the α7 subunit of the acetylcholine receptor expressed on macrophages that controlled systemic TNF release (153). Since nerve fibers in the spleen lack the enzymatic machinery for acetylcholine production, systemic inflammation recruits vagus-primed T cells from the intestine to the spleen, which produce acetylcholine and mount the innate immune response (154). Other data suggest that the anti-inflammatory properties of cholinergic neurons also attenuate inflammation and injury during experimental pancreatitis and hepatitis (155, 156). In a mouse model of pancreatitis, pretreatment with the nicotinic receptor antagonist mecamylamine resulted in more severe pancreatitis increasing edema, plasma hydrolases, and IL-6 levels. Conversely pre-treatment with the selective α7nAChR agonist anabaseine strongly decreased the severity of pancreatitis suggesting that there may be a therapeutic role of the “cholinergic anti-inflammatory pathway” in the treatment of acute pancreatitis in order to attenuate inflammation and injury (155). As a matter of fact that cholinergic neurons have a systemic anti-inflammatory effect, vagotomy increased mortality in mice upon Fas-induced hepatitis whereas pretreatment with nicotine or α7nAChR agonist, inhibited this detrimental effect of vagotomy and rescued the mice (156).

### Rheumatoid Arthritis

Rheumatoid arthritis is the most common inflammatory arthritis and affects up to 1.25% of the entire population (157). The pathogenesis is multidimensional and includes a genetic predisposition in addition to environmental challenges leading to synovial inflammation and eventually resulting in bone erosions, cartilage damage and eventually joint deformities and disabilities (158). Recent advances in the understanding of autoimmune diseases such as rheumatoid arthritis uncovered a pivotal role of the autonomous nervous system in disease pathogenesis (159). It has been shown that treatment with α7nAChR agonist improved arthritis scores in animal models of rheumatoid arthritis whereas α7nAChR knock-out mice showed worse disease outcome suggesting its therapeutic potential (Figure 4B) (160, 161). In fact, work provided by Koopmann and colleagues showed that electric stimulation via an implantable vagus nerve-stimulating device inhibits the production of TNF, IL-1β, and IL-6 and improved clinical scores of rheumatoid arthritis in patients (9). Together with similar data obtained in asthmatic patients, this study provides a proof-of-concept that treatment via activation of the cholinergic anti-inflammatory pathway is effective and may translate into the regular clinical setting.

## Summary and Future Perspective

Preclinical studies targeting neuro-immune interactions upon stimulation of the vagus nerve, application of acetylcholine agonist, and β2 adrenoreceptor agonists have emerged the potential successful treatment in inflammatory diseases (155, 162, 163). Of note, the site specific control of immune functions by the nervous system via neurotransmitters/neuropeptides suggest that the nervous system can exert a rapid and local control of immune cells. Unlike the systemic effects of cytokines, neuronal regulation of immune responses allows for the selective and spatiotemporal control of immune functions without affecting the activity of distant cells. Based on this assumption, targeting neuro-immne interactions might allow for specific and targeted therapy at a cellular and compartmental level. The therapeutic potential of neuronal modulation of inflammation in humans was already demonstrated by stimulating the vagal nerve with electronic devices that has been successfully used for the treatment of rheumatoid arthritis and asthma (9, 164). Of note, some patients did no longer respond to a conventional anti-inflammatory treatment but developed disease improvement upon vagal nerve stimulation (9). Another pilot study showed the efficacy of vagal nerve stimulation in patients with Crohn's disease whereas its stimulation improved inflammatory parameters and clinical symptoms (141). This work provides a rationale for the potential of modulating neuro-immune interactions and shows promising results reflecting that vagal-nerve stimulation may be an alternative to pharmacological therapies. This observation is further supported by a clinical study that has shown asthma improvement during non-invasive vagal nerve stimulation (164). Current study enrolments of patients with a broad variety of diseases highlight the particular interest in neuro-immune interactions [Post-surgery Systemic Inflammation and Neuro-immune Interactions (POSINI) NCT03055325, Vagal Nerve Stimulation for Gastroparesis (VNS) NCT0312 NCT03908073, Transcutaneous VNS to Treat Pediatric IBD (STIMIBD) NCT03863704]. The increase of chronic inflammatory diseases in Western societies with a significant amount of non-responders to current treatment strategies underlines the need to uncover novel strategies/medications. Therefore, it is crucial to improve our understanding of how neurons interact with immune cells. Recent technical advances, such as the RiboTag system, imaging tools, genetic mouse models built the rationale to mechanistically understand neuronal-immune circuits in more detail and further uncover signaling pathways that could be therapeutically harnessed (165–168).

## Author Contributions

MJ and CK wrote the manuscript. SM designed figures, contributed significantly to the study and the writing of the article.

### Conflict of Interest

The authors declare that the research was conducted in the absence of any commercial or financial relationships that could be construed as a potential conflict of interest.
